# Candidate malaria susceptibility/protective SNPs in hospital and population-based studies: the effect of sub-structuring

**DOI:** 10.1186/1475-2875-9-119

**Published:** 2010-05-08

**Authors:** Nahid A Eid, Aymen A Hussein, Abier M Elzein, Hiba S Mohamed, Kirk A Rockett, Dominic P Kwiatkowski, Muntaser E Ibrahim

**Affiliations:** 1 Department of Molecular Biology, Institute of Endemic Diseases, Medical Campus, Qasser Street, University of Khartoum, Khartoum, Sudan; 2 Wellcome Trust Centre for Human Genetics, University of Oxford, Roosevelt Drive, Headington, Oxford, UK; 3 Wellcome Trust Sanger Institute, Wellcome Trust Genome Campus, Hinxton, Cambridge. UK

## Abstract

**Background:**

Populations of East Africa including Sudan, exhibit some of the highest indices of genetic diversity in the continent and worldwide. The current study aims to address the possible impact of population structure and population stratification on the outcome of case-control association-analysis of malaria candidate-genes in different Sudanese populations, where the pronounced genetic heterogeneity becomes a source of concern for the potential effect on the studies outcome.

**Methods:**

A total of 72 SNPs were genotyped using the Sequenom^® ^iPLEX Gold assay in 449 DNA samples that included; cases and controls from two village populations, malaria patients and out-patients from the area of Sinnar and additional controls consisting of healthy Nilo-Saharan speaking individuals. The population substructure was estimated using the Structure 2.2 programme.

**Results & Discussion:**

The Hardy-Weinberg Equilibrium values were generally within expectation in Hausa and Massalit. However, in the Sinnar area there was a notable excess of homozygosity, which was attributed to the Whalund effect arising from population amalgamation within the sample. The programme STRUCTURE revealed a division of both Hausa and Massalit into two substructures with the partition in Hausa more pronounced than in Massalit; In Sinnar there was no defined substructure. More than 25 of the 72 SNPs assayed were informative in all areas. Some important SNPs were not differentially distributed between malaria cases and controls, including SNPs in *CD36 *and *NOS2*. A number of SNPs showed significant p-values for differences in distribution of genotypes between cases and controls including: rs1805015 (in *IL4R1*) (*P *= 0.001), rs17047661 (in *CR1*) (*P *= 0.02) and rs1800750 *(TNF-376)*(*P *= 0.01) in the hospital samples; rs1050828 (*G6PD+202*) (*P *= 0.02) and rs1800896 (*IL10-1082*) (*P *= 0.04) in Massalit and rs2243250 (*IL4*-589) (*P *= 0.04) in Hausa.

**Conclusions:**

The difference in population structure partly accounts for some of these significant associations, and the strength of association proved to be sensitive to all levels of sub-structuring whether in the hospital or population-based study.

## Background

Malaria is one of the major causes of mortality among children worldwide and is thus one of the strongest known forces for evolutionary selection in the recent history of humans. This is demonstrated by the numerous signatures of selective pressure in the genome including some of the most common polymorphisms. In fact resistance factors for malaria were first discovered half a century ago, largely as a result of Haldane's insight that malaria was the likely evolutionary driving force behind common erythrocyte variants in tropical populations [1].

The past decade has seen growing evidence of ethnic differences in susceptibility to malaria and of the diverse genetic adaptations to malaria that have arisen in different populations [1]. Differences in susceptibility to malaria have been observed between populations in malaria-endemic areas; and the fact that there might be a genetic basis for these differences has been demonstrated repeatedly in studies of sympatric populations that share the same environment but suffer different levels of malaria infection and disease burden[2,3]. Such population differences in susceptibility to malaria are becoming more relevant to modern approaches of mapping genes [4] of susceptibility/protection from malaria, understanding the epidemiology of the disease and its potential control including vaccination. The fact that different malaria-resistance alleles have arisen in different places suggests that a great deal of selection by malaria has happened relatively recently in human history and certainly following humans migration out of Africa [5].

The Sudan is a country that occupies a central position for African populations and is a host for some of the most outstanding genetic variations in the continent [5,6] with anticipated corresponding differences in traits of health and disease. In order to address the possible impact of an ensuing population structure and stratification on the outcome of case-control studies in different populations, the malaria genotype data for candidate SNPs from population cohorts in eastern Sudan, as well as from a hospital-based study from an endemic area in central Sudan, were analysed for the presence of such potential impact.

## Methods

### Study design

This is both a longitudinal and cross-sectional study of the population and hospital-based case-control studies. The study was approved by the Ethical Committee of the Institute of Endemic Diseases, University of Khartoum; Samples were taken with written informed consent from all individuals.

Malaria status was established by microscopic examination of thick and thin blood films for asexual *Plasmodium falciparum *parasites for all members of two villages, and also in the hospital-based samples. An Immune Chromatography Test [ICT] was also done as a rapid diagnostic test for malaria and confirmation of diagnosis.

### Study population

The study consists of two epidemiological settings: one a hospital-based study in which samples were obtained both from in-patients diagnosed with malaria from major hospitals in the Sinnar area, and outpatients in a health center of displaced populations in an area known as Abyai. The other setting is population-based where two populations (Hausa and Massalit in Koka and Um-Salala villages respectively) were sampled. These populations reside in the malaria-endemic area of the Rahad river in Eastern Sudan, and have been followed-up over the past decade for different infectious diseases including malaria. Information on malaria infection is based on both cross-sectional surveys that takes place once or twice a year and also on continuous passive-case detection over the past five years.

### Marker genotyping and statistical analysis

A total of 72 SNPs were genotyped using the Sequenom^® ^iPLEX Gold assay in 449 DNA samples from the two populations in eastern Sudan, in addition to a cohort of malaria patients from hospitals in Sinnar area and a control sample set consisting of healthy Nilo-Saharan speaking individuals. The SNPs were chosen based on previously published reports of malaria candidate-gene associations in addition to SNPs that has shown early promise for associations in a genomewide study undertaken in the Gambia by the MalariaGEN consortium[4]http://www.MalariaGEN.net.

Allele frequency, genotype distribution as well as departure from Hardy-Weinberg Expectations (DHWE) were calculated for all SNPs with minor allele frequency of > 0.1 (informative SNPs).

Statistical analysis for the differences in distribution of genotypes between malaria cases and controls was carried out using Fisher's Exact and Chi-Squared Tests for trend only, as the limitation of sample size did not allow testing for other models. The population substructure was estimated using the programme STRUCTURE 2.2. The STRUCTURE parameters were 10,000 burn-in periods and 10,000 step chains with up to 5 populations assumed (K1-K5), 10 replicates were run for each *K*. The maximum *K *value was determined on the basis of the likelihood distribution reaching a maximum.

## Results

### Population structure

There was DHWE in the Hausa for one SNP and for three SNPs in the Massalit out of the 26 informative SNPs (Table 1). However, DHWE was observed in a total of eight SNPs in Sinnar area (Table 2). There was a notable excess of homozygosity in six of these SNPs (rs1803632 [GBP2], rs1801033 [C6], rs2706384 [IRF1], rs10775349 and rs2230739 [ADCY9], rs3092945 and rs1126535 [CD40LG]) while in two SNPs (rs1800750 [TNF] and rs229587 [SPTB]) there was an excess of heterozygotes H = 0.932 and 0.618 respectively (Table 3), consistent with values of the *F *statistics (Table 2). In the Rahad populations (Hausa and Massalit) there was generally no difference in heterozygosity, both groups displaying normal to slightly deficient values (Table 2). Koka village contained fewer families (83) compared to Um-Salala (185). The Former village characterized by a large number of individuals per family, while in contrast Um-Salala showed a smaller number of individuals per family (see Additional file 1).

**Table 1:** The rs numbers, chromosomal position, gene symbol of the SNPs analysed in this study.

rs number	Alternate name	Chromosome	Position	Gene Symbol
rs1803632		1	89582690	GBP7
rs1800896	IL10-1082	1	206946897	IL10
rs17047661	CR1-Swain-Langley	1	207782889	CR1
rs17561	IL1A G4845T	2	113537223	IL1A
rs708567		3	9960070	IL17RE
rs4833095		4	38799710	TLR1
rs1801033		5	41199959	C6
rs2706384		5	131826880	IRF1
rs2243250	IL4-589	5	132009154	IL4
rs1800750	TNF-376	6	31542963	TNF
rs8176747		9	136131315	ABO
rs8176746		9	136131322	ABO
rs229587		14	65263300	SPTB
rs2230739		16	4033436	ADCY9
rs10775349		16	4079823	ADCY9
rs1805015	IL4R-63011	16	27374180	IL4R
rs8078340	NOS2-1659	17	26129212	NOS2
rs8386		20	57485812	GNAS
rs3092945	CD40L+220	X	135729609	CD40LG
rs1126535	CD40L-726	X	135730555	CD40LG
rs1050829	G6PD+376	X	153763492	G6PD
rs1050828	G6PD+202	X	154111023	G6PD

SNP information taken from Ensembl release 56 http://www.ensembl.org and NCBI36, dbSNP130 http://www.ncbi.nlm.nih.gov

Alternate name refers to previously published nomenclature.

**Table 2:** Hardy-Weinberg Equilibrium test values for SNPs in the Study Populations of Hausa, Massalit, and the hospital sample in Sinnar.

*Areas*	rs1803632	rs1801033	rs2706384	rs1800750	Rs3092945	rs1126535	rs229587	rs4833095	rs8176747	rs8176746
**HWE Sinnar**	0.038	0.02	0.023	2.00E+14	0.012	0.038	0.043	0.005	NS	NS
***F *Statistics**	0.226	0.237	0.154	0.002	0.433	-0.761	-0.237	0.263	ND	ND
**HWE Massalit**	NS	NS	NS	NS	0.045	NS	NS	NS	0.026	0.028
**HWE Hausa**	NS	NS	NS	NS	0.0026	NS	NS	NS	NS	NS

*F *Statistic was performed for the Sinnar sample to quantify the direction of the marked observed deviation.

*NS = Non significant *p value; *ND = Not done

**Table 3:** Expected and observed heterozygosity values for SNPs deviating from Hardy-Weinberg Expectations in the study populations.

Area	Name	ObsHET	PredHET	HWpval
**Sinnar (N = 58)**	rs1803632	0.388	0.499	0.0386
	rs17047661	0.213	0.296	0.0207
	rs2706384	0.34	0.456	0.0231
	rs1800750	0.932	0.498	2.12E-15
	rs229587	0.618	0.498	0.0428
	rs10775349	0.39	0.496	0.0462
	rs3092945	0.333	0.486	0.0124
	rs1126535	0.141	0.235	0.0023

**Massalit (N = 87)**	rs3092945	0.377	0.5	0.0452
	rs8176747	0.188	0.285	0.0262
	rs8176746	0.19	0.288	0.0282

**Hausa (N = 66)**	rs3092945	0.266	0.446	0.0026

The number of tested individuals (N).

**Figure 1:**
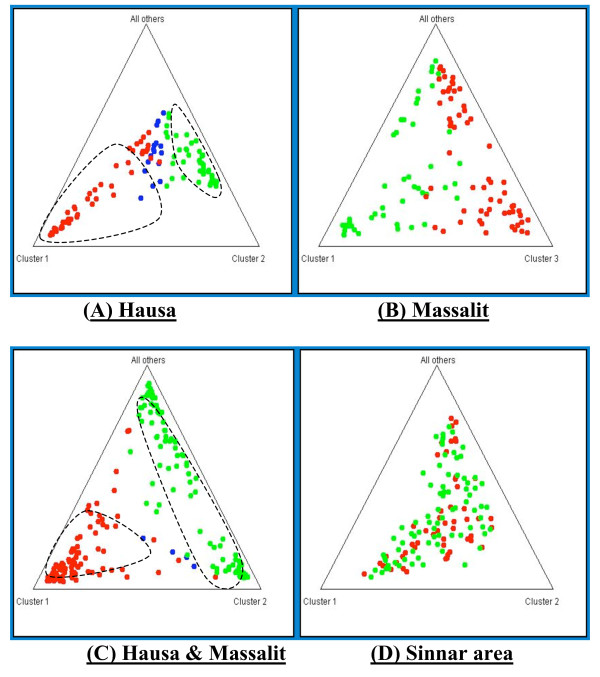
**Triange Plots produced by the program Structure for the genotyped SNPS in Rahad Area (A, B & C), and Sinnar area (D).** Dotted lines defines the conspicuous clusters.

The programme STRUCTURE revealed the division of both Hausa and Massalit into two substructures, the substructure in Hausa was more defined into two compact clusters, while in the Massalit the clustering was less-well defined. When Hausa and Massalit were entered as if they were one population in the programme input file, two conspicuous clusters corresponding to the village populations were found, one of which (Hausa) was more compact than the other. In Sinnar (hospital study), the structure result did not resolve any substructure neither between those of different ethnic background nor between cases and controls (Figure 1).

### Genotype and allelic association results

In the three areas (Koka, Um-Salala and Sinnar) a total of 26 SNPs were informative (frequency > 0.1). SNP genotypes in some known malaria candidate genes were not differentially distributed between malaria cases and controls, including SNPs in CD36, which were monomorphic in Massalit.

In the hospital-based study (Sinnar area), there were significant differences in allele frequency for a limited number of SNPs (often with direct impact on association.). For Example, the difference in genotype distribution between cases and controls in the Abyai area for rs8386 (GNAS) (*P *= 0.004) and rs1800750 (*P *< 2.2e-16) (Table 4), was due to both SNPs having minor allele frequencies of 0.08 in the cases, while in the controls (Nilotics samples) the minor allele frequencies were 0.27, 0.37 for rs8386 and rs1800750 respectively (Additional file 2).

**Table 4:** Malaria candidate-genes SNP associations based on case-control analysis in the two study sites (Sinnar and Rahad).

SNPs	Hausa vs control (N = 60/57)	Hausa + Massalit vs controls (N = 120/126)	Massalit vs Control (N = 60/69)	Abyay vs Nilotics (N = 67/53)	Sinnar Cases vs Controls (N = 125/78)	Sinnar vs combined (N = 58/78)	Sinnar vs Nilotics (N = 58/53)
rs1050829	NS	NS	p = 0.02 (0.17)	NS	NS	NS	NS
rs1050828	NS	NS	p = 0.04 (0.048)	NS	NS	NS	NS
rs1800750	NA	NA	NA	p < 2.2 × 10^-16^ (6.17 × 10^-23^)	*P *= 3.145 × 10^-13^ (4.9 × 10^-24^)	NS	NS
rs3092945	P = 0.05 (0.049)	P = 0.0025 (0.0074)	NS	p = 0.0091 (0.007)	0.013	*P *= 0.01 (0.35)	p = 0.01 (0.5)
rs1805015	NS	P = 0.0036 (0.032)	NS	P = 0.025 (0.022)	*P *= 0.065 (0.0080)	*p *= 0.001 (0.068)	p = 0.00018 (0.0044
rs2243250	p = 0.04	NS	NS	NS	NS	NS	NS
rs17561	NS	NS	NS	NS	p = 0.019 (0.156)	NS	NS
rs708567	NS	NS	NS	p = 0.022 (0.021)	NS	NS	*P *= 0.022
rs1800896	NS	p = 0.036 (0.23)	p = 0.04 (0.28)	NS	NS	NS	NS
rs17047661	NS	NS	NS	NS	NS	p = 0.02 (0.89)	p = 0.00045 (0.0009)
rs8386	NA	NA	NA	p = 0.0040 (0.0021)	NS	NS	NS
rs10775349	NA	NA	NA	p = 0.013 (0.012)	p = 0.01216 (0.0034)	NS	NS
rs2230739	NA	NA	NA	p = 0.016 (0.0067)	NS	NS	NS
rs8078340	NA	NA	NA	p = 0.019 (0.016)	NS	NS	NS

Association is calculated for the combined samples and for the sub-samples in each site. *P *values estimated at the 95% significance for the general trend and in a genotype interactive model (Bracketed). NS = Non Significant *P *Value; NA = results not available for either SNP not tested or test failure.

rs17047661 was the only polymorphism to differ significantly in allele frequency between the hospital and population samples. The allele frequency for the minor allele was 0.44 in the hospital samples, while in the population samples the frequency was 0.74 and 0.73 in Massalit and Hausa respectively (Additional file 2).

In the hospital-based study highly significant values for differences in genotype distribution were initially found when control samples were included from our data (Nilotics) for three SNPs: rs1805015 (0.00018), rs1126535 (0.015) and rs17047661 (0.00045). However, upon the inclusion of additional control samples from hospital outpatients with negative malaria, one SNPs lost its significance altogether (rs708567 [IL17R]), two SNPs gave lower significance (rs1805015, and rs17047661), while rs1126535 maintained the same significance (*P *= 0.015) (Table 4). In rs1805015 the CC genotype was found at a low frequency in cases compared with controls (Additional file 3) (*P *= 0.001, OR = 3.96). This is the only SNP where the odds ratio inverted almost consistently across the sample sets (OR = 3.2-7.68.) (Additional file 4) Interestingly, the effect seems to differ between the Sinnar and Rahad area, implying a risk influence in Sinnar versus protection in the Rahad. Likewise, the case with SNPs in the Abyai area: rs8386 (*P *= 0.0040) rs2230739 (*P *= 0.016), rs8078340 [NOS2] (*P *= 0.019), rs708567 (*P *= 0.022), where significance was attained only in the subsample with exception of the rs10775349 where significance was in both the combined and subsample. In the combined sample from the Sinnar area (125 cases vs 78 controls), three SNPs became significant: rs1800750 (*P *< 2.2 × 10^-16^) in Abyai and (*P *= 3.145 × 10^-13^) in the combined sample; rs1126535 (*P *= 0.0091) in Abyai and (*P *= 0.013) in the combined sample; rs1805015 (*P *= 0.025) in Abyai and (*P *= 0.065) in the combined sample. These three SNPs showed significant associations in both the Abyai and combined samples, although significance decreased in the combined sample in a manner similar to inclusion of hospital controls with rs1126535.

In the population-based study, genotype frequencies were differentially distributed in Massalit between cases and controls in three SNPs with *P *values of *P *= 0.02, (rs1050829 [G6PD]), *P *= 0.04 (rs1050828) and (rs1800896 [IL10]) (Table 4), although the later has lost its significance upon correction in a genotype interactive model. In Hausa differences in genotype distribution was found only in two SNPs. The rare CC homozygote for rs2243250 was not found in cases, while it was found in 6/6 controls (See Additional file 5) (P = 0.04), and rs1126535 as stated above (*P *= 0.05) (Table 3). When the two populations were pooled, one SNP became significant for association (rs1805015 *P *= 0.0036), rs1800896 maintained its significance as in Massalit while the rs1800750 became highly significant upon pooling (*P *= 0.0025) (Table 4).

Malaria distribution by population substructure was analysed using the programme STRUCTURE 2.2. In Hausa malaria was equally distributed between the two clusters, 43% and 45% in cluster 1 and 2 respectively. While in Massalit where only one major cluster (cluster 3) was identified, 86.5% of the total malaria prevalence was in this cluster (Table 5). Table 6 shows the effect of removing individuals from the minor clusters on the P values of association before and after reanalysis.

**Table 5:** Population clusters in the Hausa and Massalit villages based on the program STRUCTURE (Figure 1C), and the distribution of malaria cases within the different clusters.

Area	Status	Cluster 1	Cluster 2	Cluster 3
**Hausa**	Malaria	22(43%)	23(45%)	N/A
	No malaria	20(39%)	22(43%)	N/A

**Massalit**	Malaria	4(8%)	1(2%)	32(41%)
	No malaria	5(10%)	5(10%)	46(59%)

Cluster 3 is non-existent in Hausa (N/A).

**Table 6:** Effect of population sub-structuring on malaria association, the change in the *P *Value for the same SNPs before and after exclusion of the minor clusters in Massalit.

	P value	
SNPs	Before	After
**rs1800896**	0.04	0.066
**rs1805015**	0.1	0.05
**rs1126535**	0.06	0.033
**rs1050828**	0.02	0.02

## Discussion

Recent genomic studies have produced detailed genome wide descriptions of genetic diversity and population structure for a wide variety of human populations [7,8]. The outcomes of this information however are yet to be fully employed in trait mapping and association studies; this is particularly crucial within the African context where the population genetic structure and patterns of LD seems to bear profound impact on such studies.

In the present study, two sets of case-control data are compared: a study undertaken at a hospital and outpatient clinic in central Sudan, and a population sample from two ethnic groups (villages) in Eastern Sudan. These sets were analysed independently for sub-structuring since due to some minor differences in the panel of 72 SNPs used.

The population structure included: the two main ethnic groups Hausa and Massalit who were classified according to language and ethnicity, but also defined by the programme STRUCTURE. Interestingly the outcome of the STRUCTURE analysis supported the differences of the two groups based on patterns of genotypes for 72 SNPs of mostly unlinked loci. In the hospital-based study, the structure considered malaria inpatients from Sinnar hospital and inpatients and outpatients controls in addition to patients from a local clinic. The control samples were individuals of Nilotic origin from our local database. The relevance of population structure was manifested in the current set of data all the way from stratification of hospital case-control samples to the combined village population, down to the village sub-structures.

Using DHWE, heterozygosity and STRUCTURE to compare and contrast population structure, it was noted that the impact of such structure on the degree of association was most pronounced in the hospital-based study where the inclusion of a control from a different ethnic group resulted in classically inflated values of significance with spurious associations in three out of five positive associations. The rs1126535 had the most striking *P *value and DHWE, The fact that association occurs across the study sites suggests that this is not an outcome of differences in minor allele frequencies, as no such difference was observed between sites and populations. Although disparities in allele frequency make an ideal source of spurious association, they should not be always discarded, as differences in functional alleles of this sort might explain varied response between populations in susceptibility/protection from disease.

The effect of the population ethnic background was also shown in the fact that some of the SNPs reported to associate with malaria susceptibility/protection in West Africa, such as those in CD36 [9,10], had not shown any association in this study and were non-polymorphic altogether in one population (Massalit). The programme STRUCTURE was also used as a tool for stratification where it was evident that with such a limited sets of SNPs it was possible to cluster the village populations. When populations were combined and input into STRUCTURE, the programme assigned the two populations into two separate clusters; one (Hausa) being more defined than the other. This is perhaps due to the fact that the Hausa, with their extended families, widely practiced polygamy and higher percentage of within-village marriages seems to be more endogamous than Massalit [11]. Structure also partitioned each Hausa and Massalit into two substructures, although this was not justified in terms of Fst or in the programme output values, which indicate that the candidate genes used in the analysis, with their adaptive non-neutral nature and limited numbers of SNPs are probably not the best population differentiation markers.

There is no evidence of departure from random mating in the two major sample sets and the DHWE seen here may be due to subtle effects of sub-structuring and the contribution of several populations with varying allele frequencies. This was particularly pronounced in the Sinnar sample and the fact that most of the DHWE were due to an excess of homozygosity or what is known as the Whalund effect resulting from population Apparently DHWE for the rs1800750 SNP, which was encountered more often amalgamation [12,13], is an example of a locus-under-natural-selection possibly due to some other diseases including meningitis (a common disease in the Sahel).

An example of the effect of genetic structure on association is the significant association that was initially observed in the hospital samples for three SNPs in prominent candidate genes: TNF, IL4, and CR1. The hits were more significant when using a control sample from another population (Nilotics); the use of additional control samples from the same area led to a decrease in the significance of association but not its disappearance. The decrease in significance was partly due to differences in minor allele frequencies that are often encountered in populations of different ethnic backgrounds, which was also reflected in the DHWE in the combined sample as aforementioned. Interestingly, only one SNP had lost its significance of association altogether, indicating that the SNPs concerned might authentically be involved in malaria pathology. Those SNP associations with severe malaria are well known and are commonly reported in the literature [14]: CR1 [15], IL4 [16,17], TNF [18], G6PD[19], IL10 [20]. However, no single SNP was found to be associated with protection/susceptibility across areas and populations with the exception of rs1126535 in CD40LG (OR = 2.4 in the Rahad to 5.4 in Sinnar), and the rs1805015 in the IL4 receptor (OR = 0.41 in Rahad to 7.6 in Sinnar), such a disparate range of values and functions could not be explained at the present and with the limited sample set.

The population substructure of the Hausa had no significant impact on the malaria distribution since malaria was equally distributed between clusters; however in the Massalit who mostly segregated into one major cluster, the removal of the minor cluster altered the significance of association. Although this might be an outcome of a small sample size, the variation of association between the different sample combinations in an analytical context still needs to be put into perspective, in order to explain why a *P *value is maintained or lost.

The prevalence of malaria in Koka and Um-Salala villages, during all cross sectional surveys and extensive follow up over the past 5-10 years, was found to be different between the two villages; clinical malaria of uncomplicated nature being more common in Um-Salala than Koka whereas infection with asymptomatic malaria was higher in Koka. Such differences of malaria status is speculated to be due to differences in the immune response an outcome of the difference in the genetic structure, which is in turn could be attributed to the genetic history and ethnic variations between populations as seen in West Africa[1,21]. The relationship of the sickle-cell mutation in the haemoglobin gene (sickle), population structure and malaria in these villages is reported elsewhere [11]. The analysis of the interaction and combined effect of sickle with some of these polymorphisms might prove interesting for an in-depth understanding of the overall mechanism of malaria susceptibility/protection in these populations.

In the present study, it was possible to implicate with a fairly modest sample size a few candidate genes in malaria protection/susceptibility and to establish a role for population stratification. The issue of sample size and power attainment is one of the main challenges facing genomewide analysis and trait mapping. The complexity of performing such analysis on populations with diverse genetic backgrounds and pronounced population structure was highlighted in a recent study by the MalariaGEN Consortium [4] where a sample of a reasonable size could not detect with satisfactory power a common polymorphism with major effect under the high stringency of multiple testing. The authors suggested an imputation approach based on sequence information to address the problem. The issue of detecting rare variants with possible major effect such as rs2243250 in this study is paramount since obviously such a SNP will have no chance of passing the stringency of multiple testing in a genomewide analysis even with approved sample size. This issue also brings into focus the issue of epidemiological studies in village or populations of limited sizes (~1,000) where an adequate number of independent genotypes might be rather difficult to establish.

## Conclusion

With a limited set of SNPs and a rather modest population size for a case-control study, the effects of population structure on the outcome of such studies was clearly demonstrable. This is relevant to situations where the population size of a village is too small to allow independent genotypes, or when malaria incidence is low. The population structure is a myriad of states that include age, population size, and endogamy. The programme STRUCTURE revealed the marked contrast between the village and hospital-based samples where the latter apparently lacked any form of structure that could be revealed by the current set of SNPs. The structure in the village is apparently a function of both the unified ethnicity and relatedness of the population (familial).

In Sinnar, where controls were selected from a population that was previously characterized based on estimates of Fst value, the analysis resulted in an inflated number of hits as compared to using a locally matched control.

However, although allele frequency difference is a potential source for spurious association, it should not be always discarded. The divergent evolutionary histories per locus, underlying such allele difference, may often be shaped by related infectious diseases that shares related biological mechanisms, hence explaining major differences between populations for disease profiles.

## Competing interests

The authors declare that they have no competing interests.

## Authors' contributions

NAE Collected the samples and analysed the data and contributed to the writing of manuscript. AH analysed the data and contributed to the manuscript writing. AME carried out the QC genotyping. HSM contributed to the analysis and laboratory work. K R, contributed to data analysis and manuscript revision; DK in charge of the overall genotyping and QC setting, revised the manuscript. MEI conceived of the study supervised the analysis and wrote the manuscript. All authors approved the final manuscript

## Supplementary Material

Additional file 1 Total number of family units per village, and distribution of malaria in Koka and Um-Salala villages per family unit. Red bars (Salala), blue bars (Koka). Click here for file

Additional file 2 Allele frequencies of SNPs that showed differences in distribution between malaria cases and controls in all studied samples. Click here for file

Additional file 3 Genotype and allele frequencies of SNPs displaying differences in distribution between malaria cases and controls in the hospital sample. Click here for file

Additional file 4 **Odds ratio for the SNPs presenting with significant association in the case control analysis of all study populations.** Confidence intervals (bracketed). The values in bold employs a general model, while the plain text uses a logistic regression model. Accepted values are underlined. Click here for file

Additional file 5 Genotype and allele frequencies of SNPs that had differences in distribution between Malaria cases and controls in Hausa and Massalit. Click here for file
