# Supplementary material for: Candidate malaria susceptibility/protective SNPs in hospital and population-based studies: the effect of sub-structuring
Source: Malar J. 2010 May 8;9:119. doi: 10.1186/1475-2875-9-119 (PMC2877684; doi:10.1186/1475-2875-9-119)
Supplement: Additional file 1 — Total number of family units per village, and distribution of malaria in Koka and Um-Salala villages per family unit. Red bars (Salala), blue bars (Koka). [file 1475-2875-9-119-S1.DOC]

**Additional file 2: Allele frequencies of SNPs that showed differences in distribution between malaria cases and controls in all studied samples**

*NA= results Not available either to test failure or other reason

| **SNPs** | **Allele** | **Abyai** | **Nioltics** | **Sinnar** | **Hausa** | **Massalit** |
| --- | --- | --- | --- | --- | --- | --- |
| rs1126535 | C | 0.45 | 0.75 | 0.5 | 0.38 | 0.5 |
|  | T | 0.55 | 0.25 | 0.5 | 0.62 | 0.5 |
| rs1805015 | C | 0.32 | 0.52 | 0.27 | 0.48 | 0.31 |
|  | T | 0.68 | 0.48 | 0.73 | 0.52 | 0.69 |
| rs17047661 | A | 0.39 | 0.24 | 0.56 | 0.27 | 0.26 |
|  | G | 0.61 | 0.76 | 0.44 | 0.73 | 0.74 |
| rs2243250 | C | 0.21 | 0.26 | 0.29 | 0.24 | 0.17 |
|  | T | 0.79 | 0.74 | 0.71 | 0.76 | 0.83 |
| rs1050829 | C | 0.23 | 0.22 | 0.2 | 0.2 | 0.24 |
|  | T | 0.77 | 0.78 | 0.8 | 0.8 | 0.76 |
| rs1800896 | C | 0.31 | 0.44 | 0.42 | 0.35 | 0.49 |
|  | T | 0.69 | 0.56 | 0.58 | 0.65 | 0.51 |
| rs10775349 | G | 0.29 | 0.51 | 0.51 | NA | NA |
|  | C | 0.71 | 0.49 | 0.49 | NA | NA |
| rs708567 | A | 0.57 | 0.35 | 0.46 | 0.4 | 0.45 |
|  | G | 0.43 | 0.65 | 0.54 | 0.6 | 0.55 |
| rs1800750 | A | 0.08 | 0.63 | NA | NA | NA |
|  | G | 0.92 | 0.37 | NA | NA | NA |
| rs8386 | C | 0.92 | 0.73 | NA | NA | NA |
|  | T | 0.08 | 0.27 | NA | NA | NA |
| rs2230739 | A | 0.91 | 0.94 | NA | NA | NA |
|  | G | 0.09 | 0.06 | NA | NA | NA |
| rs8078340 | C | 0.81 | 0.88 | NA | NA | NA |
|  | T | 0.19 | 0.12 | NA | NA | NA |
