# Supplementary material for: Candidate malaria susceptibility/protective SNPs in hospital and population-based studies: the effect of sub-structuring
Source: Malar J. 2010 May 8;9:119. doi: 10.1186/1475-2875-9-119 (PMC2877684; doi:10.1186/1475-2875-9-119)
Supplement: Additional file 2 — Allele frequencies of SNPs that showed differences in distribution between malaria cases and controls in all studied samples. [file 1475-2875-9-119-S2.DOC]

**Additional file 3: Genotype and allele frequencies of SNPs displaying differences in distribution between malaria cases and controls in the hospital sample.**

| **rs1805015 C/T** | **Genotypes Frequency** | | | **Allele Frequency** | |
| --- | --- | --- | --- | --- | --- |
|  | **CC** | **CT** | **TT** | **C** | **T** |
| Malaria cases | 1 | 27 | 26 | 0.37 | 0.63 |
| Controls | 16 | 26 | 22 |  |  |
| *P* | 0.001 | | | NS | |
| **rs3092945 *C/T*** | **CC** | **CT** | **TT** | **C** | **T** |
|  |  |  |  |  |  |
| Malaria cases | 15 | 18 | 13 | 0.6 | 0.4 |
| Controls | 31 | 8 | 14 |  |  |
| *P* | 0.01 | | | NS | |
| **rs17047661 *A/G*** | **AA** | **AG** | **GG** | **A** | **G** |
|  |  |  |  |  |  |
| Malaria cases | 11 | 24 | 17 | 0.54 | 0.46 |
| Controls | 27 | 22 | 11 |  |  |
| *P* | 0.02 | | | NS | |

*NS= Non significant *p value*
