# Supplementary material for: Candidate malaria susceptibility/protective SNPs in hospital and population-based studies: the effect of sub-structuring
Source: Malar J. 2010 May 8;9:119. doi: 10.1186/1475-2875-9-119 (PMC2877684; doi:10.1186/1475-2875-9-119)
Supplement: Additional file 3 — Genotype and allele frequencies of SNPs displaying differences in distribution between malaria cases and controls in the hospital sample. [file 1475-2875-9-119-S3.DOC]

**Additional file 4: Odds ratio for the SNPs presenting with significant association in the case control analysis of all study populations. Confidence intervals (bracketed). The values in bold employs a general model, while the plain text uses a logistic regression model. Accepted values are underlined.**

| **SNPs** | **Hausa + Massalit vs controls** (N=120/126)  **Odds Ratio** | | | **Sinnar Cases vs controls** (N= 125/78)  **Odds Ratio** | | |  |
| --- | --- | --- | --- | --- | --- | --- | --- |
|  | **Hausa vs control**  **Odds Ratio** |  | **Massalit vs Control**  **Odds Ratio** | **Abyay vs Nilotics**  **Odds Ratio** | | **Sinnar vs Combined**  **Odds Ratio** | **Sinnar vs Nilotics**  **Odds Ratio** |
| rs1050829 | NS | NS | 0.32(0.08 -1.24) | NS | NS | NS | NS |
| rs1050828 | NS | NS | 0.38(0.13-1.15) | NS | NS | NS | NS |
| rs1800750 | NA | NA | NA | **1.03 (0.18-5.79)**) | **3.81(1.08-13.40)**) | NS | NS |
| rs1126535 | **2.81(0.96-8.16**) 1.01(0.37-2.72) | **2.41(1.20-4.81)** 0.98(0.49-1.95) | NS | **1.67(0.51-5.42)** 5.4(1.74-16.71) | **1.58(0.76-3.27)** 4.32(2.08-8.99) | **0.76 (0.15-3.86)**  0.45 (0.12-1.65) | **1.14(0.17-7.60)** 0.58(0.14-2.43) |
| rs1805015 | NS | **0.41(0.18-0.90**) 0.76(0.33-1.71) | NS | **1.04(0.36-3.01)** 3.33(1.16-9.59) | **1.57(0.74-3.35)** 3.02(1.41- 6.46) | **3.03(0.89-10.37)** 3.96(1.14-13.74) | **4.97(1.39-17.82)** 7.68 (2.04-28.87) |
| rs2243250 | NA | NS | NS | NS | NS | NS | NS |
| rs17561 | NS | NS | NS | NS | **1.95(0.98-3.89)** 1.33(0.18-9.66) | NS | NS |
| rs708567 | NS | NS | NS | **0.53(0.19-1.52**) 0.24(0.08-0.69) | NS | NS | NS |
| rs1800896 | NS | **0.59 (0.28-1.25)** 0.50(0.23-1.12) | **0.61(0.24-1.51**) 0.42(0.14-1.23) | NS | NS | NS | NS |
| rs17047661 | NS | NS | NS | NS | NS | **0.82(0.38-1.76)** 0.82(0.23-2.86) | **0.35(0.09-1.44)** 0.10(0.026-0.43) |
| rs8386 | NA | NA | NA | **0.25(0.09-0.69)** | NS | NS | NS |
| rs10775349 | NA | NA | NA | **0.61(0.24-1.55)** 0.19(0.06-0.60) | **0.77(0.42-1.41)** 0.31(0.15- 0.63) | NS | NS |
| rs2230739 | NA | NA | NA | **8.67(1.06-70.77)** | NS | NS | NS |
| rs8078340 | NA | NA | NA | **3.88(1.46-10.30**) 1.23(0.16-9.28) | NS | NS | NS |
