# Supplementary material for: Candidate malaria susceptibility/protective SNPs in hospital and population-based studies: the effect of sub-structuring
Source: Malar J. 2010 May 8;9:119. doi: 10.1186/1475-2875-9-119 (PMC2877684; doi:10.1186/1475-2875-9-119)
Supplement: Additional file 4 — Odds ratio for the SNPs presenting with significant association in the case control analysis of all study populations. Confidence intervals (bracketed). The values in bold employs a general model, while the plain text uses a logistic regression model. Accepted values are underlined. [file 1475-2875-9-119-S4.DOCX]

**Additional file 5: Genotype and allele frequencies of SNPs that had differences in distribution between Malaria cases and controls in Hausa and Massalit**

| **Massalit**  **rs1800896** | **Genotypes Frequency** | | | **Allele Frequency** | |
| --- | --- | --- | --- | --- | --- |
|  | **CC** | **CT** | **TT** | **C** | **T** |
| Malaria cases | 16 | 24 | 11 | 0.49 | 0.51 |
| Controls | 11 | 32 | 18 |  |  |
| P | 0.04 | | | NS | |
| **rs1050829** | **CC** | **CT** | **TT** | **C** | **T** |
|  |  |  |  |  |  |
| Malaria cases | 11 | 7 | 32 | 0.24 | 0.76 |
| Controls | 6 | 12 | 43 |  |  |
| P | 0.02 | | | NS | |
| **rs1050828** | **CC** | **CT** | **TT** | **C** | **T** |
|  |  |  |  |  |  |
| Malaria cases | 22 | 2 | 3 | 0.92 | 0.08 |
| Controls | 57 | 5 | 1 |  |  |
| P | 0.04 | | | NS | |
| **Hausa**  **rs2243250** | **CC** | **CT** | **TT** | **C** | **T** |
| Malaria cases | 0 | 22 | 33 | 0.24 | 0.76 |
| Controls | 6 | 18 | 29 |  |  |
| P | 0.04 | | | NS | |
| **rs3092945** | **CC** | **CT** | **TT** | **C** | **T** |
|  |  |  |  |  |  |
| Malaria cases | 10 | 24 | 21 | 0. | 0. |
| Controls | 14 | 12 | 29 |  |  |
| P | 0.05 | | | NS | |

*NS= Non significant *P value*
